# N-terminal pro-brain natriuretic peptide: a potential follow-up biomarker of mandibular advancement device efficacy on cardiac function in obstructive sleep apnea

**DOI:** 10.12688/f1000research.17081.2

**Published:** 2019-03-05

**Authors:** Denis Monneret

**Affiliations:** 1 Department of Biochemistry and Molecular Biology, South Lyon Hospital Group, Hospices Civils de Lyon, Pierre-Bénite, 69495, France

**Keywords:** N-terminal pro-brain natriuretic peptide, mandibular advancement device, obstructive sleep apnea, cardiac biomarker

## Abstract

Interrelationships between obstructive sleep apnea (OSA) and cardiovascular diseases are now recognized, but some underlying pathophysiological mechanisms remain controversial. Circulating cardiac biomarkers are diagnostic tools that can help understand them, in particular the N-terminal pro-brain natriuretic peptide (NT-proBNP), a marker of myocardial stretch, and a potential indicator of subclinical cardiac stress in OSA. Continuous positive airway pressure (CPAP), the first-line treatment of moderate to severe OSA, may be considered as uncomfortable, resulting in poor adherence, and reduced effectiveness. In this case, mandibular advancement devices (MAD) are an effective alternative therapy, more comfortable, and generally well accepted, with higher compliance. To date, few studies have compared the cardiovascular effects of CPAP and MAD. From recent literature reviews, it emerges that both therapies are effective in blood pressure reduction. However, the effects of MAD on other cardiovascular outcomes are conflicting, in particular as regards to its impact on circulating cardiac biomarkers. In a recent ancillary study from a randomized controlled trial, Recoquillon *et al* concluded that two months of MAD treatment had no effect on NT-proBNP plasma levels in patients with severe OSA. The present discussion analyses this result from a biological, statistical, and analytical standpoint, in light of results from other studies evaluating natriuretic peptides in MAD-treated OSA, with the aim to support further longitudinal studies designed with a high methodological quality.

Mandibular advancement devices (MADs) are an effective alternative to continuous positive airway pressure (CPAP) in the treatment of obstructive sleep apnea (OSA). OSA is associated with increased cardiovascular morbidity and mortality, and an increasing number of studies highlight the efficacy of MADs in terms of both sleep apnea, and cardiac outcomes ^1– 3^. Unlike CPAP-related studies, few studies to date have focused on cardiac biomarkers under MAD therapy in OSA.

In a recent randomized controlled trial, Recoquillon *et al.* evaluated the effect of two months of MAD treatment on N-Terminal Pro-Brain Natriuretic Peptide (NT-proBNP) plasma levels in patients with severe OSA ^4^. Compared to a sham device, the high treatment adherence (6.6 hours/night) significantly reduced the mean apnea-hypopnea index (AHI), and the oxygen desaturation index. Nevertheless, according to their model, the authors stated that MADs had no effect on NT-proBNP levels, nor on other inflammatory and metabolic biomarkers. To our knowledge, to date only two studies have investigated the natriuretic peptides in such contexts ^5, 6^. Given their scarcity, any type of study looking at relevant cardiac biomarkers of MAD efficacy must be encouraged, and designed in as detailed and robust a manner as possible. In this way, some issues have to be discussed regarding the evaluation of NT-proBNP from Recoquillon *et al*.

*1) Biological standpoint.*

After two months of MAD use, the NT-proBNP plasma concentrations decreased from 296.8 to 252.5 pg/mL (–14.9%) in treated patients, whereas they decreased from 189.8 to 184.3 pg/mL (–2.9%) in patients with the sham device, resulting in a mean adjusted intergroup difference of 12.0 pg/mL (–40.9 to 64.9, 95%CI; *P* =0.65). The question arises as to whether a NT-proBNP decrease of about 15% after treatment is biologically significant. Indeed, according to the specifications of the desirable biological variation database ^7^, this decrease should be considered as significant according to the within-subject biological variation (CVi 10%), but not significant according to the between-subject biological variation (CVg 16%). In any case, this decrease should be considered as analytically significant since it exceeds the allowable limit of total error, which combines the analytical imprecision and the inter-method inaccuracy, fixed at 13% for NT-proBNP. Moreover, one could argue that a longer treatment period, even one extra month, could be sufficient to significantly lower its circulating level. In support of this assumption, Hoekema *et al*. showed a significant decrease in NT-proBNP (‒58%, *P* =0.035) in ten patients with moderate to severe OSA treated by MAD (adherence 6.8 hours/night, 6.9 nights/week) after a period of 69 to 82 days ^5^. For these ten patients, baseline (52 pg/mL, interquartile range (IQR): 13‒105), and follow-up NT-proBNP values (22 pg/mL, IQR: 15‒33) were within or close to the reference intervals established according to the method ^8^, and were thus in accordance with exclusion criteria discarding patients with a history of cardiovascular disease (CVD). Unlike Hoekema *et al*., NT-proBNP values from Recoquillon *et al*. reached 500 to 700 pg/mL, *i.e.* much higher than the normal values announced by the manufacturer (median 47 pg/mL, min–max: 3.9–155 pg/mL ^9^). This is somewhat in contradiction with the exclusion criteria supposed to discard patients with a history of CVD, including heart failure ^4^. Another study showed a significant decrease of plasma BNP levels (‒24%) after 6 months of MAD therapy in patients with stable, mild to moderate congestive heart failure (CHF), and OSA ^6^. Although less stable in plasma than NT-proBNP ^10^, BNP, the other biomarker of CHF, is still widely and routinely assayed on analyzers in hospital laboratories, and thus remains of potential interest for the follow-up of cardiac function under MAD treatment.

*2) Statistical standpoint.*

In the supplemental statistical section, Recoquillon *et al*. mentioned that variables with non-continuous distributions are described as median (IQR) ^4^. However, NT-proBNP results were expressed as mean (standard deviation (SD)), and reached 296.8 (401.6) pg/mL. Such a wide SD suggests a strong skewness of distribution, with possibly the presence of extreme outliers at baseline and/or at follow-up, in the low values and/or in the high values, which could ultimately false the observed difference in concentrations. The impact of outliers is well-known in research, be they of clinical or laboratory origin ^11, 12^; they must be detected through appropriate methods, possibly with adjustment for skewness ^13^, and their presence requires investigation, especially for small groups. In the study from Recoquillon *et al.*, a median (IQR) expression would therefore have been more appropriate than mean (SD), and a graph detailing the scatter dot plots for both groups, with connecting lines before and after treatment, would have been required. Moreover, a linear regression analysis was used for the adjustment of baseline values and potential covariates: age, gender, body mass index, and baseline AHI. Nevertheless, these covariates were used for the adjustment of all biomarkers, but no statistical proof was provided as regards to their degree of correlation with NT-proBNP specifically. Furthermore, given the limited number of patients (n ±55), if NT-proBNP results were not normally distributed (as seems to be the case), nonparametric ANCOVA or robust regression methods would probably have been more appropriate ^14– 17^.

*3) Analytical standpoint.*

Recoquillon *et al*. assayed plasma NT-proBNP using a multiplex electrochemiluminescent immunoassay on a MESO QuickPlex® SQ120 analyzer (MSD, Rockville, USA). Using this technology for assaying this cardiac biomarker is somewhat unusual. Indeed, as reminded by the manufacturer, this method is for research use only, but not for use in diagnostic or therapeutic procedures. It involves three incubation steps, interspersed with three wash sequences, requiring at least five hours of preparation for one 96-well plate. To our knowledge, no studies based on this assay have been published up to now, not even the eight references cited in the MSD technical sheet of the human NT-proBNP assay kit ^9^. Given the long and tedious assay protocol impractical in hospital laboratory routine, and given the absence of hindsight about its analytical performance, the authors should rather have used a most widespread, reliable, and rapid automated method, like the electrochemiluminescent immunoassay method on Roche analyzers (Roche Diagnostics, Mannheim, Germany) ^18^, which was, moreover, available in the laboratory from one of the five hospital centers participating to the trial (Poitiers Hospital Center ^19^, NCT01426607 ^20^).

The strong association between the severity of OSA and a higher prevalence and incidence of cardiovascular events has been evidenced for a long time ^21^. However, where cardiac biomarkers are useful for the diagnosis of many cardiovascular diseases, the interest of natriuretic peptides in the evaluation of OSA-associated cardiac dysfunction and CPAP effects remain controversial, especially because of uncontrolled co-morbidities, as summarized by Maeder *et al.* ^22, 23^. To date, no study has specifically evaluated whether NT-proBNP reflects cardiac dysfunction in OSA or whether NT-proBNP is under the influence of obesity in this context.

An interesting perspective is the ongoing MOSAIC study, whose main objective is to assess the impact of three months of MAD therapy on AHI in Asian patients with heart failure and OSA ^24^. Indeed, one of the planned secondary objectives is the evaluation of cardiac remodeling and of cardiac biomarkers, including NT-proBNP. Meanwhile, the present standpoints remind and emphasize the need for close collaborations between sleep specialists and laboratory practitioners to strengthen the methodological quality and robustness of studies involving biomarkers.

## Data availability

All data underlying the results are available as part of the article and no additional source data are required.
