# Supplementary material for: N-terminal pro-brain natriuretic peptide: a potential follow-up biomarker of mandibular advancement device efficacy on cardiac function in obstructive sleep apnea
Source: F1000Res. 2019 Mar 5;7:1818. Originally published 2018 Nov 20. [Version 2] doi: 10.12688/f1000research.17081.2 (PMC6415324; doi:10.12688/f1000research.17081.2)
Supplement: Peer review history

# Peer review: Reviewer response for version 2

**DOI:** 10.5256/f1000research.20154.r45262

**Published:** 2019-03-12

**Authors:** Micha Maeder

**Affiliations:** 1 Division of Cardiology, Cantonal Hospital St. Gallen, St. Gallen, Switzerland

The authors has appropriately revised the manuscript. I have no further comments.

I have read this submission. I believe that I have an appropriate level of expertise to confirm that it is of an acceptable scientific standard.

# Peer review: Reviewer response for version 1

**DOI:** 10.5256/f1000research.18676.r43027

**Published:** 2019-01-31

**Authors:** Micha Maeder

**Affiliations:** 1 Division of Cardiology, Cantonal Hospital St. Gallen, St. Gallen, Switzerland

The present manuscript is a relatively extensive comment on a short original manuscript (brief communication) published in Thorax. The key finding of the paper by Recoquillon et al. ^1^ was a lack of effect of effective OSA treatment by mandibular advancement therapy on NT-proBNP. This finding is in the centre of the comment by Denis Monneret.

 From my point of view the result was far away from being statistically significant, and the observed (non-significant) difference may have been the result of chance. Therefore, I cannot fully follow the argument listed under “biological standpoint”. The key problem however is the fact that it is currently unknown what BNP and NT-proBNP reflect in patients with OSA. The literature on this issue has been summarized in detail by Maeder MT et al. in Clin Chim Acta (2016) ^2^, and the overall summary is that the data is conflicting. No study has in detail evaluated whether NT-proBNP in OSA reflects cardiac dysfunction or whether NT-proBNP is mainly under the influence of obesity in OSA. Variable effects of CPAP on BNP and NT-proBNP in uncontrolled studies have been observed. In the present manuscript I would really like the author to mention these aspects.

 With regards to the statistical standpoint I fully agree that BNP and NT-proBNP always display a skewed distribution, and that this should have been taken into account. When using non-parametric tests, the result is even more unlikely to be statistically significant.

 With regards to the analytical standpoint I also agree: the use of an established easy to use NT-proBNP assay would have made it easier to check the plausibility of the data and to compare it with the existing literature.

I have read this submission. I believe that I have an appropriate level of expertise to confirm that it is of an acceptable scientific standard, however I have significant reservations, as outlined above.

## References

[1] RecoquillonSPépinJLVielleBAndriantsitohainaRBironneauVChouet-GirardFFleuryBGoupilFLaunoisSMartinezMCMeslierNNguyenXLParisAPriouPTamisierRTrzepizurWGagnadouxF: Effect of mandibular advancement therapy on inflammatory and metabolic biomarkers in patients with severe obstructive sleep apnoea: a randomised controlled trial. *Thorax*.2018; 10.1136/thoraxjnl-2018-212609 10.1136/thoraxjnl-2018-212609 30366971

[2] MaederMTSchochODRickliH: A clinical approach to obstructive sleep apnea as a risk factor for cardiovascular disease. *Vasc Health Risk Manag*.2016;12: 10.2147/VHRM.S74703 85-103 10.2147/VHRM.S74703 27051291PMC4807890

# Peer review: Reviewer response for version 1

**DOI:** 10.5256/f1000research.18676.r42053

**Published:** 2019-01-17

**Authors:** Gen-Min Lin

**Affiliations:** 1 Department of Medicine, Hualien Armed Forces General Hospital, Hualien, Taiwan

In general, the comments were written well, as the cited paper is a randomised control trial design and the author made a critical appraisal. Since many readers might not have time to review the original paper, it might be helpful to write a summary before the comments. Otherwise no further comments were made; and the report is suitable to be accepted in current version.

I have read this submission. I believe that I have an appropriate level of expertise to confirm that it is of an acceptable scientific standard.
